# Methodology for analysis and reporting patterns of failure in the Era of IMRT: head and neck cancer applications

**DOI:** 10.1186/s13014-016-0678-7

**Published:** 2016-07-26

**Authors:** Abdallah S. R. Mohamed, David I. Rosenthal, Musaddiq J. Awan, Adam S. Garden, Esengul Kocak-Uzel, Abdelaziz M. Belal, Ahmed G. El-Gowily, Jack Phan, Beth M. Beadle, G. Brandon Gunn, Clifton D. Fuller

**Affiliations:** 1 Head and Neck Section, Division of Radiation Oncology, Department of Radiation Oncology, The University of Texas M.D. Anderson Cancer Center, Box 0097, 1515 Holcombe Blvd, Houston, TX 77030 USA; 2 Department of Clinical Oncology and nuclear medicine, Faculty of Medicine, Alexandria University, Alexandria, Egypt; 3 Department of Radiation Oncology, Case Western Reserve University, Cleveland, OH USA; 4 Department of Radiation Oncology, Beykent University, Istanbul, Turkey; 5 Graduate School of Biomedical Science, University of Texas Health Science Center, Houston, TX USA

**Keywords:** Patterns of failure, IMRT, Head-and-neck cancer, Deformable image registration

## Abstract

**Background:**

The aim of this study is to develop a methodology to standardize the analysis and reporting of the patterns of loco-regional failure after IMRT of head and neck cancer.

**Material and Methods:**

Twenty-one patients with evidence of local and/or regional failure following IMRT for head-and-neck cancer were retrospectively reviewed under approved IRB protocol. Manually delineated recurrent gross disease (rGTV) on the diagnostic CT documenting recurrence (rCT) was co-registered with the original planning CT (pCT) using both deformable (DIR) and rigid (RIR) image registration software. Subsequently, mapped rGTVs were compared relative to original planning target volumes (TVs) and dose using a centroid-based approaches. Failures were then classified into five types based on combined spatial and dosimetric criteria; A (central high dose), B (peripheral high dose), C (central elective dose), D (peripheral elective dose), and E (extraneous dose).

**Results:**

A total of 26 recurrences were identified. Using DIR, recurrences were assigned to more central TVs compared to RIR as detected using the spatial centroid-based method (*p* = 0.0002). rGTVs mapped using DIR had statistically significant higher mean doses when compared to rGTVs mapped rigidly (mean dose 70 vs. 69 Gy, *p* = 0.03). According to the proposed classification 22 out of 26 failures were of type A (central high dose) as assessed by DIR method compared to 18 out of 26 for the RIR because of the tendencey of RIR to assign failures more peripherally.

**Conclusions:**

RIR tends to assigns failures more peripherally. DIR-based methods showed that the vast majority of failures originated in the high dose target volumes and received full prescribed doses suggesting biological rather than technology-related causes of failure. Validated DIR-based registration is recommended for accurate failure characterization and a novel typology-indicative taxonomy is recommended for failure reporting in the IMRT era.

**Electronic supplementary material:**

The online version of this article (doi:10.1186/s13014-016-0678-7) contains supplementary material, which is available to authorized users.

## Introduction

Intensity-modulated radiation therapy (IMRT) is one of the most important innovations in modern radiation therapy and represents a paradigm shift in the treatment of head and neck cancers (HNCs). However, there are certain hazards that may increase the risk of loco-regional failure (defined as tumor persistence or recurrence) including inadequate definition of the tumor extension and clinically important target volumes (TVs), uncertainties related to daily positioning, weight loss or deformation of tumor and normal tissues during the course of treatment, and uncertainties in plan optimization, dose calculation and treatment delivery [1–5].

The accurate and specific definition of the exact site of failure, in addition to the radiation dose given to this site is, therefore, mandatory to identify the possible cause(s) of failure. The classic definition of failures as “local”, or “regional”, was appropriate in the setting of conventional radiotherapy using large homogeneous dose-volumes, but is no longer helpful nor descriptive of distinct types of failure in patients treated with IMRT [6–8].

Several previous efforts have addressed the importance of studying the patterns of failure after IMRT treatment of HNCs, [2, 4, 9–13] with most reporting failures as “infield”, “marginal” or “outfield” based on the percentage of overlap between the failure volume and the respective TV on the treatment planning CT (pCT) [4, 9, 10, 12, 13].

The ability to accurately describe the relation of failure to original TVs and dose mandates a fairly precise method to co-register the diagnostic CT documenting recurrence (rCT) to the original pCT. However, the majority of the previous studies implemented mainly rigid image registration techniques (RIR) [2, 4, 10, 12, 13]. RIR is simple, quick and widely used but it allows only for 6° of freedom and doesn’t account for changes in the shapes or relative positions of different regions-of-interests (ROIs) [14]. Emerging data demonstrate the superiority of deformable image registration (DIR) compared to RIR in registering pCT to on-treatment CT or conebeam CT in the setting of image guided radiotherapy (IGRT) for HNCs [15–17]. However, very few studies addressed DIR software implementation for the purpose of registering the diagnostic rCT to the original pCT [6, 7].

Our group has recently validated different registration techniques used for co-registering diagnostic contrast enhanced head and neck CT to non-contrast planning CT and showed DIR was superior for this application [18]. As a continuation of these efforts and to validate DIR as a tool to improve accurate definition of the patterns of loco-regional failure in the era of IMRT for HNCs, we sought to undergo the following specific aims:

1. Develop a workflow methodology to standardize the analysis of HNCs patterns of failure using both geometric and dosimetric parameters.
2. Assess the impact of registration (rigid vs. deformable) techniques on patterns of failure quantitative analytic parameters.
3. Develop a granular classification and nomenclature to optimize the accurate reporting of distinct failure typology.

## Material and methods

### Patients

Tumor registry data for patients diagnosed with head and neck squamous cell carcinoma, whom were treated by IMRT at The University of Texas, MD Anderson Cancer Center between 2006 and 2009, were retrospectively reviewed under an institutional review board approval. 600 patients were identified, of those 103 had a documented recurrence. A total of 21 cases were randomly selected from the recurrence dataset based on the following eligibility criteria: IMRT given for curative intent; treatment of intact tumor (i.e. post-operative cases were excluded); equal distribution of various head and neck subsites (i.e. nasopharynx, oropharynx, hypopharynx, and lateral neck “i.e. neck nodes of unknown primary site”); radiological evidence of local and/or regional failure; available CT scan of failure site prior to any salvage therapy; and pathologic and/or radiologic evidence of recurrence (i.e. biopsy, or high SUV on PET).

### IMRT treatment planning and delivery

All patients had been positioned supine in an individualized thermoplastic head and shoulder mask for CT simulation and treatment and a custom dental stent used as an intraoral immobilization device [19, 20]. A treatment pCT scan was used for defining TVs. Target volume definition was done in Pinnacle treatment planning system (Pinnacle, Phillips Medical Systems, Andover, MA), with rigorous multi-physician target delineation and quality assurance [21, 22].

Treatment was uniformly delivered using Varian (Varian Medical Systems, Palo Alto, CA) linear accelerators delivering 6-MV photons. Three clinical target volumes (CTVs) were typically defined. CTV definitions and dose prescriptions are summarized in Additional file 1: Table S1. Treatment was delivered in a conventional fractionation scheme (average 33 fractions). Patients were treated using a monoisocentric technique with an antero-posterior low-neck supraclavicular field matched to the IMRT fields or using whole neck IMRT for cases where gross nodes are located at the match line.

### Post-treatment follow up

Initial post-treatment evaluations were made at 8–12 weeks after therapy completion and subsequently every 2–3 months for the first year, every 3–4 months for the second year, and at least twice a year up to 5 years.

### Loco-regional failure

Cases where local and/or regional recurrence was recorded had their immediate post-failure diagnostic images exported as DICOM files from the clinical PACS system to the treatment planning system, where radiological evident recurrent gross disease (rGTV) was manually contoured by a radiation oncologist (ASRM) and reviewed by a head and neck service-specific attending radiation oncologist (CDF).

### Image registration

For each patient, the rCT or rPET-CT was co-registered with pCT using both rigid and deformable image registration techniques. DIR was performed using a commercial software (ADMIRE, Elekta AB, 2015) validated previously by our group for the registration of contrast-enhanced diagnostic CT to non-contrast enhanced planning CT [18]. Deformation vector fields were obtained from DIR algorithm, mapping the deformation of the rCT onto the pCT. Subsequently, in a custom written Matlab routine (MATLAB R2013a, The MathWorks Inc., Natick, MA, 2013), pCT; dose grid; original plan TVs; rCT; and rGTVs were imported. The deformation fields were then applied to rGTVs segmented on the rCT to convert them into ‘deformed rGTVs’ on the pCT.

**Fig. 1:**
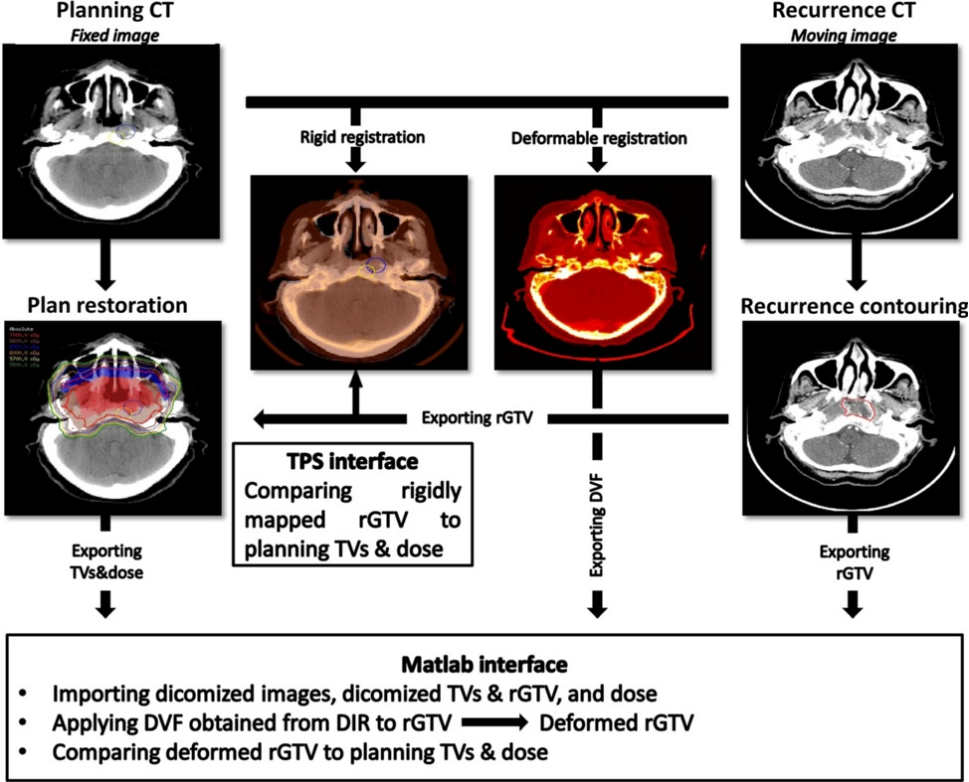
Workflow process of patterns of loco-regional failure registration process

Evaluation of deformed rGTVs relative to original planning TVs was done using both centroid-based method that assumed the center of mass of rGTV was the origin of the recurrence volume and its location was compared relative to planning TV after applying deformation vector fields (DVF). Simultaneously, RIR was performed using the rigid co-registration tool available in the Pinnacle planning system to rigidly align rCT to pCT, following that rGTVs where exported to patient’s plan where dose volume histograms (DVHs) and rGTV centroids were generated and analysis metrics were calculated. Figure 1 illustrates the workflow process of our registration methodology.

### Analysis of failure metrics

For both RIR and DIR mapped rGTVs the following metrics were evaluated:

1) Recurrence volume, 2) Location of the centroid relative to planning TV: Centroid is the central voxel of the recurrence volume plus added 2 mm margin to account for registration error, 3) Spatial relationship of rGTV centroids to IMRT/supraclavicular match line and ipsilateral parotid in case of peri-parotid failure, and 4) Mean and maximum dose to rGTVs, dose to 95 % failure volume (fD95%), and mean dose to centroid volume.

### Classification of failure

**Fig. 2:**
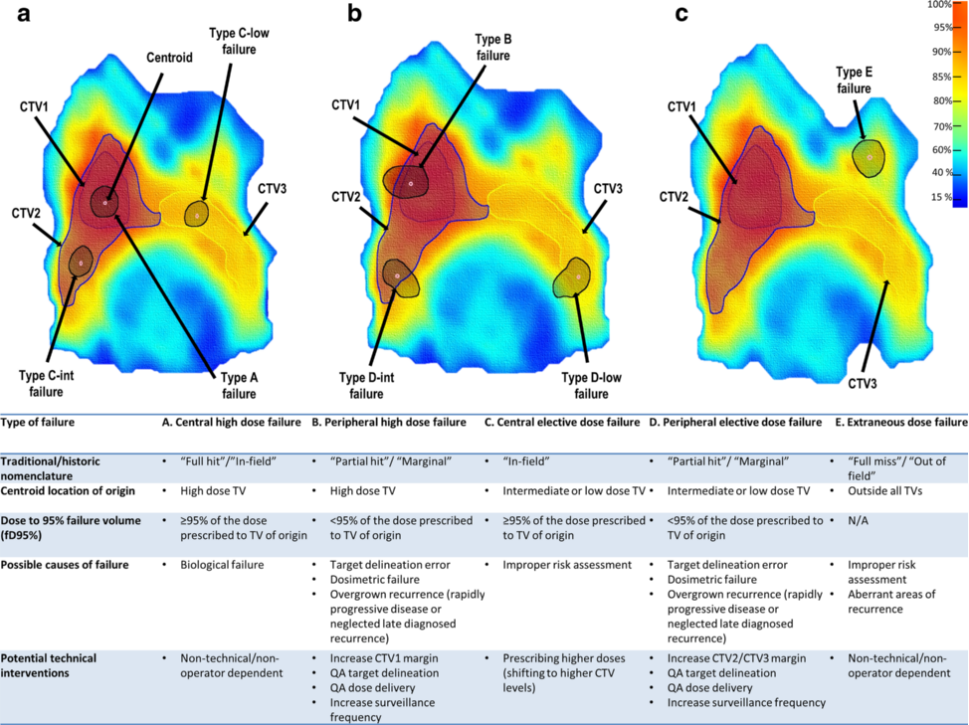
Classification scheme of IMRT patterns of failure using combined centroid based geometric method coupled with the dosimetric parameters. Panel **a**) shows an example of types A (central high dose) and C (central elective dose) failures, panel **b**) shows an example of types B (peripheral high dose) and D (peripheral elective dose) failures, and panel **c**) shows an example of type E (extraneous dose) failure.

In order to refine our reporting and quality assurance practices using a standard nomenclature, we developed a granular typology of failure categories relative to the planning TV and dose. As illustrated in Fig. 2, failures were classified into five types based on combined spatial and dosimetric criteria:

- Type A: Central high dose failure, where the mapped failure centroid originates in high dose TV and the dose to 95 % failure volume (fD95%) is ≥95 % dose prescribed to corresponding TV of origin.
- Type B: Peripheral high dose failure, where the mapped failure centroid originates from high dose TV but its fD95% receives <95 % dose prescribed to this TV.
- Type C: Central elective dose failure, where the failure centroid originates from elective dose TV and its fD95% receives ≥95 % dose prescribed to the respective TV.
- Type D: Peripheral elective dose failure, where the failure centroid originates from elective dose TV and its fD95% receives <95 % dose prescribed to the respective TV.
- Type E: Extraneous dose failure, where the failure centroid originates outside all TVs.

For patients treated with low-neck supraclavicular field matched to the IMRT fields, two additional types were added:

- Type F: Junctional failures at the site of IMRT/supraclavicular match line.
- Type G: Low neck failures at the site of low-neck supraclavicular field.

### Statistical analysis

Non parametric statistics were used to compare analysis metrics for centroid locations and dosimetric parameters of failures mapped using RIR versus DIR registration techniques. A *p*-value ≤ 0.05 was deemed significant. Statistical assessment and data tabulation was performed using JMP v 11Pro (SAS institute, Cary, NC).

## Results

### Patients

A total of 21 patients with HNSCC were included in this pilot methodology/workflow development study. Median age was 58 years (range 30–75), and 86 % were men. Patient, disease, and treatment characteristics are presented in Table 1. Recurrences were delineated using diagnostic contrast-enhanced CT in 16 patients and using PET-CT in 5 patients.

**Table 1:** Patient demographics, disease, and treatment characteristics

	Total	
	*n* = 21	(%)
Age (years)		
Median	58	
Range	30–75	
Time to Failure (months)		
Median	12	
Range	5–69	
Sex		
Male	18	(86)
Female	3	(14)
Origin		
Nasopharynx	6	(28)
Oropharynx	5	(24)
Hypopharynx	5	(24)
Unknown primary	5	(24)
T-category		
T0	5	(24)
T1	1	(5)
T2	7	(33)
T3	5	(24)
T4	3	(14)
N-category		
N0	1	(5)
N1	5	(24)
N2	12	(57)
N3	3	(14)
Treatment		
Radiation alone	4	(19)
Concurrent ChemoRadiation	9	(43)
Induction Chemotherapy + Radiation	1	(5)
Induction Chemotherapy + Concurrent ChemoRadiation	7	(33)
Radiation dose		
Mean (SD)	69.2	(1.7)
Radiation fractions		
Mean (SD)	33	(2)

### Spatial/dosimetric failure mapping

#### Spatial mapping

A total number of 26 rGTVs were delineated. Mean rGTVs volume was 12.5 cm^3^ (range 1–105). The registration method independently affected the spatial location of mapped failures. Failures mapped using DIR were significantly assigned to more central TVs compared to failures mapped using RIR. 38 % of centroids (n = 10) mapped using RIR were located peripheral to the same centroids mapped using DIR (*p* = 0.0002). Table 2 illustrates the sites and geometric details of all failures mapped to the pCT.

**Table 2:** Geometric details of failed rGTVs

	n.	Percent
N. of recurrences	26	
Recurrence volume		
Mean (SD)	12.5	(23)
Location of centroid using RIR		
GTV	12	(46)
CTV1	7	(27)
CTV2	1	(4)
CTV3	1	(4)
PTV1	4	(15)
Supraclavicular field	1	(4)
Location of centroid using DIR		
GTV	22	(84)
CTV1	1	(4)
CTV2	1	(4)
CTV3	1	(4)
Supraclavicular field	1	(4)

*Abbreviations:  DIR* Deformable image registration, *RR* Rigid Registration, *GTV* gross tumor volume, *CTV* clinical target volume, *PTV* planning target volume

#### Dosimetric mapping

rGTVs mapped using DIR had statistically significant higher mean doses when compared to rGTVs mapped rigidly (mean dose 70 vs. 69 Gy, *p* = 0.03) while comparison of mean fD95% was not statistically significant (mean fD95% 68 vs. 66 Gy, *p* = 0.07), and comparison of maximum, and centroid doses showed no-significant differences between both registration methods (*p* = 0.7 and 0.4, respectively). Additional file 1: Table S2 shows the dosimetric details of all failures.

### Classification of failure

**Fig. 3:**
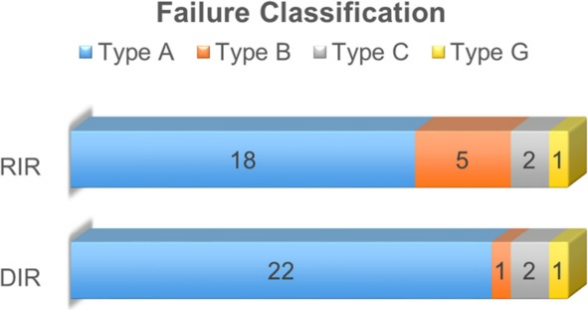
Bar chart illustrating the difference in failure classification using rigid (RIR) vs. deformable (DIR) image registration methods

Based on the proposed classification of failure using both the spatial location of the centroids of the mapped failure volumes coupled with the dosimetric parameters (as illustrated in Fig. 2), 22 (84.6 %) out of the 26 failures mapped using DIR were of type A, one of type B, 2 of type C, and one of type G. Whereas, 18 (69 %) out of the 26 failure mapped using RIR were of type A, 5 of type B, 2 of type C and one of type G. Figure 3 illustrates the difference in classification using both registration methods. There was no type F (junctional) failures in patient subset treated using anteroposterior low-neck supraclavicular field matched to the IMRT fields. Additionally, no peri-parotid failures were detected.

**Fig. 4:**
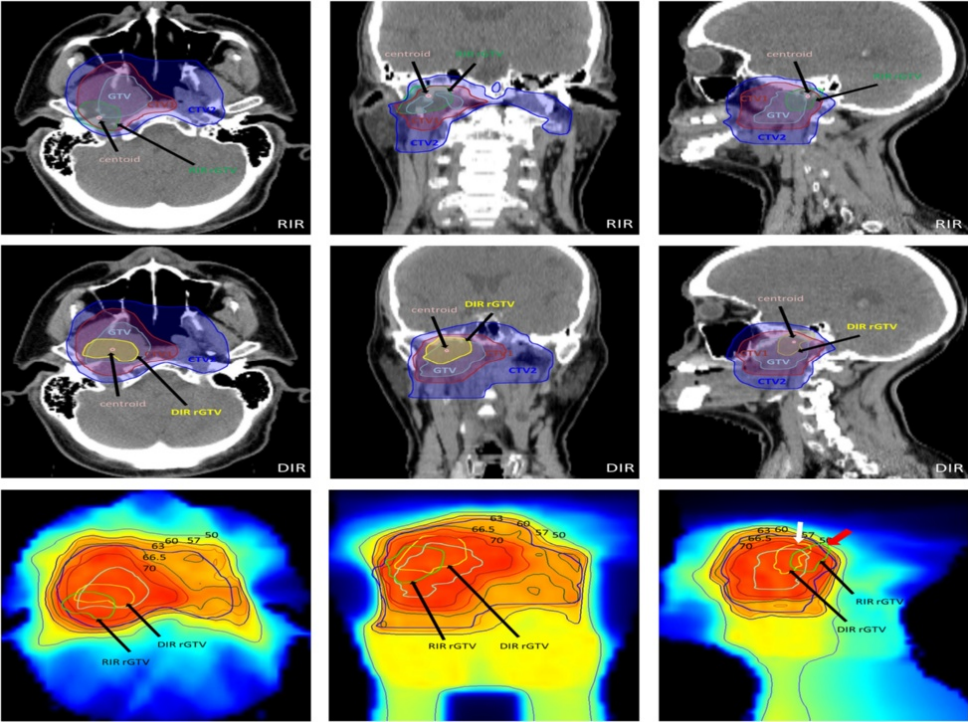
A case of T2N0 Nasopharyngeal carcinoma recurred 63 months after IMRT. The upper panel shows the axial, coronal and sagittal images of a RIR mapped rGTV on the original pCT where its centroid is located at CTV1and the 95 % rGTV volume contained on more peripheral PTV2 (contour not shown). The middle panel shows DIR mapped rGTV on the original pCT where its centroid located at GTV and the 95 % rGTV volume contained on more peripheral CTV2. The lower panel shows RIR and DIR mapped rGTVs overlaid to plan isodose line. Note that RIR rGTV fD95% extends beyond the 95 % isodose line “66.5 Gy” (*red arrow in sagittal image*) which would erroneously characterize it as type B failure, while in fact DIR shows it as a type A failure (i.e. the fD95% of DIR mapped rGTV is completely encapsulated with 95 % isodose line, shown by white arrow in sagittal image)

This combined spatial/dosimetric analysis shows that while 10 centroids (38 %) of RIR mapped rGTVs were located peripheral to the same centroids mapped using DIR as shown in Table 2. However, after adding the dosimetric component of analysis, only 4 of those 10 RIR mapped rGTVs were peripheral high dose failures (type B) and the other 6 were central high dose failure (type A) because despite the centroids were spatially peripheral in location to the respective DIR ones but dosimetrically, the rGTVs 95 % volumes still had ≥95 % dose. Figure 4 shows an example of the differences in spatial and dosimetric parameters for a DIR versus RIR mapped failure. Those 4 rGTVs were seen in the following patients: two nasopharyngeal (one primary “Fig. 4” and one nodal site); one oropharyngeal (primary site); and one unkown primary (nodal site). The secondary qualitative review by expert radiation oncologists (CDF, DIR) of those 4 patients agreed with DIR classification that those recurrences are actually central rather than peripheral in origin.

## Discussion

Traditionally, failure reporting for HNCs has simply classified disease as “local”, “regional”, or “locoregional”, thus relating location of failure to a crude anatomic reference. However, such a reporting language gives no information regarding radiation fields/volumes, or delivered dose. In the pre-IMRT era, when large fields of homogenous dose were used, the definition of “in-field” failure (i.e. within the field borders) or “marginal” failure (i.e. adjacent to the block edges) were intuitive descriptors relating treatment parameters to sites of failure. However, in the current era of conformal therapy [23, 24], dose gradients, and multiple TVs make relating spatially accurate information about dose and recurrence far more complicated for IMRT plans. In the same way that a standardized method for analysis and reporting for TVs has been undertaken successfully [23–25], a similar effort is desirable for pattern of failure reporting. In our opinion, reporting failure using only anatomic/field referents is insufficient for complex multi-volume/multi-dose plans, and obscures clinically useful information which might lead to improvements in future studies.

Likewise, the requirement of rigorous assurance for correctly localizing disease after-therapy is increased in terms of required spatial accuracy. The steep dose gradients of modern IMRT plans and proximate transition from high-risk CTVs to intermediate- or lower-risk CTVs implies inaccurate registration will erroneously assign the location of failure to incorrect dose/prescription volume. We, in fact, show RIR for IMRT plans resulted in incorrect localization relative to prior TVs and dose in 16 % of failures in the current pilot dataset. Consequently, this study presents a methodology and workflow that involves the application of quality assured DIR software as a tool to standardize co-registration and to correctly attribute sites of loco-regional failure.

Almost all previous studies have used RIR to describe the patterns of loco-regional failure after IMRT [2, 10, 12, 13]. Chao *et al.* [10] reported 17/126 loco-regional failures treated by definitive or postoperative IMRT; 53 % of failures were inside CTV1, 12 % marginal to CTV1, 6 % marginal to CTV2, and 28 % were out of field. Eisbruch et al. [2] reported on 21 recurrences in 133 patients with non-nasopharyngeal HNCs treated with parotid-sparing IMRT; 17/21 were in-field. Daly et al. [12] reported on 69 HNCs treated with parotid-sparing IMRT; 8 patients developed a loco-regional failure, 7 relapsed within the high-dose CTV, with one junctional failure observed. Sanguineti et al. [13] described the patterns of failure for 50 patients with IMRT for oropharyngeal SCC; 5 recurrences were related to high dose regions while 4 were at the low dose regions. All these reports relied on RIR, known to be less spatially accurate than DIR [18]; it is conceivable these results might be altered if DIR methods were used. Due et al. [7] reported that DIR showed slightly better reproducibility in identification of the site of recurrence origin compared to RIR. Our previous work [18], as well as the current study, confirm the qualitative superiority, in HNC applications, of DIR for CT-CT registration.

In our classification scheme, we designed a combined geometric/dosimetric typology definition to avoid the drawbacks of using each method separately. Centroid only method suppose a single point of origin and ignore the dose given to the whole area of recurrence while the dosimetric only analysis is agnostic to the geometric recurrence origin. Due et al. [7] previously reported that focal methods, such as the centroid method we used, are more accurate to localize the origins of loco-regional recurrences than volume overlap methods, which may incorrectly assign recurrences to more peripheral TVs. Raktoe et al. [8] further confirmed the superiority of focal methods like centroid expansion to the volumetric method in identifying the origin of loco-regional recurrences. The combined method we used identify the estimated site of recurrence origin relative to the respective TV in the planning CT and then compare the dose to the mapped recurrence volume with the dose prescribed to the TV of origin. Using this method, our results showed that DIR significantly assigned failures to more central TVs and doses compared to RIR concordant with the results of Due et al. [7].

Our proposed nomenclature allows granular reporting of different types of failure. In our classification, type A “central high dose” failures, are considered to be biological failures, as they likely represent resistance to maximal therapy, and thus could not conceivably be prevented by technical/operator dependant processes including IMRT QA or delineation alteration. Type A failures motivate future investigation of alternative treatment stratgies (e.g. integration of novel targeted drug therapies or dose escalation to identifiable biologically aggressive subvolumes). Likewise, type E “extraneous dose” failures cannot be modified by IMRT QA processes. They represent a possible diagnostic or decision error rather than a target delineation error (i.e. “One will never hit what one does not aim at.”). However, type B, C, and D failures are of a special concern since they entail potential technical or radiotherapy process failures. Type C “central elective (intermediate or low) dose” failures may be prevented by prescribing higher doses (i.e. shifting to higher CTV levels). Importantly, type B “peripheral high dose” or D “peripheral elective dose” failures necessitate a rigorous QA process including triple DIR registration of pre-therapy diagnostic imaging (diagnostic CT, MRI, and/or PET-CT) to pCT and the earliest rCT, to assess the potential causes: potential target delineation or dose delivery error (modifiable) versus overgrown recurrence that represents actual type A or C failure which is converted to type B or D, respectively, due to rapidly progressive disease or neglected late diagnosed recurrence (not modifiable). This involves multi-physician review of planning and recurrence contours, and review of IGRT data (i.e. set-up error, adaptive replanning datasets), as well as examination of the follow-up interval between surveillance images. By cataloging type B/D errors, we can then address the relevant issues dynamically for future patients. For instance, the only type B patient (i.e. using DIR methodology), was noted on secondary review of diagnostic imaging to have subsequent intracranial extension, route of failure, despite optimum delineation and dose coverage.

The secondary qualitative review by expert radiation oncologists (CDF, DIR) of all the clinical and imaging data of the four additional recurrences that were classified as peripheral high dose (type B) using RIR while were type A using DIR, concurred with DIR classification that those recurrences are actually central rather than peripheral in origin.

In this limited pilot dataset, our results showed the majority (84.6 %) of DIR mapped failures were of type A indicating, that biological, non-technically/non-operator dependant explanations for failure predominated. However, using RIR type A failures would have been erroneously reported as comprising only 69 %. These results assert the need for a robust, quality assured image registration technique, as error in the registration process would invalidate subsequent results and thus might deceptively indicate a greater rate of technical/operator-attributable therapy failures than DIR demonstrates. The current study, while underpowered to make clinical extrapolations due to limited number of patients, nonetheless serves as a benchmark to describe our standardized analytic and reporting method. Already, RTOG 1216, for example, contains provisions regarding collection of imaging data post-failure [26], which will allow careful analysis, and process quality improvement for future trials and large scale datasets.

## Conclusions

Rigid image registration method tends to assigns failures more peripherally compared with deformable method. Using DIR, the vast majority of failures in the presented pilot study originated in the high dose target volumes and received full prescribed doses suggesting biological rather than technology-related causes of failure. We heavily recommend a validated DIR-based registration technique in addition to granular combined geometric- and dosimetric-based failure characterization using novel typology-indicative taxonomy as a standard part of large-scale patterns of failure reporting in the IMRT era.

## Abbreviations

DIR, deformable image registration; IMRT, intensity modulated radiotherapy; IRB, institutional review board; pCT, planning CT; rCT, recurrence CT; rGTV, recurrence gross tumor volume; RIR, rigid image registration; ROIs, regions of interest; TVs, target volumes

## Additional file

Additional file 1: Table S1. IMRT target volume definitions and dose prescription. **Table S2.** Dosimetric patterns of failure. (DOCX 16 kb)
