# Supplementary material for: Methodology for analysis and reporting patterns of failure in the Era of IMRT: head and neck cancer applications
Source: Radiat Oncol. 2016 Jul 26;11:95. doi: 10.1186/s13014-016-0678-7 (PMC4962405; doi:10.1186/s13014-016-0678-7)
Supplement: Additional file 1: Table S1. — IMRT target volume definitions and dose prescription. Table S2. Dosimetric patterns of failure. (DOCX 16 kb) [file 13014_2016_678_MOESM1_ESM.docx]

**Supplementary tables**

**Supplementary table 1. IMRT target volume definitions and dose prescription.**

| Target Volume | Definition | Dose prescription |
| --- | --- | --- |
| High risk clinical target volume (CTV1) | gross tumor volume (GTV) plus margin, GTV included all known gross disease (primary tumor plus grossly enlarged lymph nodes) | 66-69.96 Gy |
| Intermediate risk clinical target volume (CTV2) | mucosal, bony, and nodal volumes at intermediate risk of harboring microscopic disease | 57-63 Gy |
| Low risk clinical target volume (CTV3) | mucosal, bony, and nodal volumes at low risk of harboring microscopic disease | 54-57 Gy |
| Planning Target Volume (PTV) | CTV plus 3-4 mm margin, with daily image guidance |  |

**Supplementary table 2.** **Dosimetric patterns of failure.**

| rGTVs dose metrics using RIR | Mean Dose in Gy. (SD) |
| --- | --- |
| Mean | 69 (6) |
| fD95% | 66 (7) |
| Max. | 71 (6) |
| Centroid | 69 (6) |
| rGTVs dose metrics using DIR |  |
| Mean | 70 (5) |
| fD95% | 68 (6) |
| Max. | 72 (5) |
| Centroid | 70 (5) |

*Abbreviations:* DIR = Deformable image registration, RIR = Rigid Image Registration, fD95%= Dose to the 95% failure volume, Max.=Maximum, SD= standard deviation.
